# Burden of Disability in a Chandigarh Village

**DOI:** 10.4103/0970-0218.40880

**Published:** 2008-04

**Authors:** Amarjeet Singh

**Affiliations:** Department of Community Medicine, Postgraduate Institute of Medical Education and Research (PGIMER), Chandigarh, India

## Introduction

Life expectancy has increased substantially in last 50 years all over the world. The pattern of disease is also shifting from acute communicable to chronic non-communicable ones. Such diseases lead to a lot of disability. More and more people with disability continue to survive as compared to previous times. Because of such a scenario, new indicators of health have been developed viz disability adjusted life expectancy (DALE) rather than traditional life expectancy. The focus is on quality of life i.e. how much of life is lived without any disability. Ten percent of the world population suffers from various disabilities (WHO).(1) In India, however, varying prevalence (1-4%)(2) has been reported. But there is lack of data on how disability affects the concerned families. Against this background, the present study was planned with an objective to ascertain the pattern and extent of disability in a Chandigarh village and to ascertain the response of affected families to such disability.

## Materials and Methods

This cross-sectional study was conducted during April 2004-March 2005 in a village selected randomly from the list of total villages (27) of Chandigarh. Of the three anganwadis in the village, one was selected randomly. Because of the resource constraints, study was confined to one village only. All the houses under the selected anganwadi were surveyed by a research fellow with the help of pre-tested/pilot-tested interview schedule. In this study, disability was considered when any routine ‘activity limitation’ was reported by the respondents.

Heads of the families were asked about the presence of any disability (locomotor, visual, auditory, mental, etc.) in the family members. The domains in the interview schedule included identification data, family profile, presence of disability, details of disability (origin, duration and severity), the response of the family to the disability, effect of disability on the daily routine life of the respondent, details of treatment sought, money spent on appliances used, community and family attitude and response as reported to the disabled person. The data were tabulated and analyzed manually using percentage, mean and chisquare test. All respondents were explained about the purpose of the study. Their consent was taken before interview. All the information was kept confidential.

## Results

Majority (70%) of the study population was Hindu. Sikhs were 26% and Muslims 4%. Majority (68%) were from lower and lower middle class. One-fourth of the houses had TV, 6% had telephone, 4% had mobile phones, 10% had fridge, 8% had scooters and 2% had a car. Literacy rate was 68%. Of the total population (1210) surveyed, 58 (4.8%) were found to have some disability. In only one of the cases, benefit of government schemes was availed. In five cases, some monetary compensation was given by the employers as these were occupational injuries (Rs. 3000-10,000). In 35 cases, the disabled persons used some appliances to overcome the disability (spectacles 15, denture 2, crutches 2, walking stick 6, hearing aid 2, artificial hand 1, belt 2, wheelchair 1 and others 4).

Disability rate was significantly more in people aged 55 years or more (31%) as compared to 5.4% in 25-54 years and 0.1% in <25-year age group (*P* < 0.001). Table 1 shows the age and sex distribution of the study population. Of the 58 disabled people, 25 had two disabilities (one had 3 and one had 4). Disability rate was significantly more in females as compared to males (*P* < 0.001). Maximum (23/58) cases of disability were visual (13 mild, 9 moderate and one severe). Eleven respondents were edentulous. Locomotion disability was seen in 12 cases (polio 3, arthritis 6 and deformed foot 3). Psychiatric/neurological disability was seen in eight cases (hemiparesis 1, paraplegia 1, mental retardation 1, schizophrenia 1, slip disc 2, spinal injury 1 and hydrocephalus 1). There were five cases of cosmetic disability (burn scar 3, dark brown spot 1 and disfigurement 1). Two deaf cases were also seen.

**Table 1:** Age and sex distribution of disability in the study population

Age (years)	Sex				Total	
		
	Male		Female			
			
	Total	Disabled	Total	Disabled	Total	Disabled
5-14	225	1	184	2	409	3
15-44	354	10	294	11	648	21
45 or more	86	8	67	26	153	34
Total	665	19 (2.85%)	545	39 (7.15%)	1210	58 (4.79%)

*x*^2^ = 12.11, df = 1, *P* < 0.001 (for sex wise prevalence). *x*^2^ = 144.69, df = 2, *P* < 0.001 (for age wise prevalence)

Twenty-three disabled people did not feel that their social or family life was affected by disability. Thirty-two people reported that some impact was there on their social life. In three cases, moderate impact on social life was told. More handicap was felt in using road, public transport or going to market. Celebration of festivals was least affected. Less handicap was felt in home and neighborhood setting.

Effect of disability on activities of daily life (ADL) is shown in Table 2. In 16 cases, only one ADL was affected. In 26 cases, 2-4 ADLs were affected and in 12 cases 5 or more ADLs were affected. Four cases reported no impact on ADL. In one case each, feeding was severely affected due to disability related to vision or intellect. Bathing was severely compromised in one mentally retarded case. It was moderately affected in five cases with locomotion disability and in three cases with visual disability.

**Table 2:** Effect of disability on activities of daily life and arrangements made by the family (n = 58)*

Activities of daily life	No. reporting limitation	Degree of limitation			Arrangements made for disability	
			
		Mild	Moderate	Severe	No	Yes
Feeding	14	8	4	2	8	6
Bathing	21	15	5	1	6	15
Transfer (Mobility)	29	19	9	1	17	12
Dressing	15	12	2	1	6	9
Toilet	19	14	5	-	11	8
Continence	2	-	-	2	-	2

* Multiple ADL were also affected in individuals

Very few respondents had consulted Government Medical College (3) or PGI (5). Fourteen had consulted private doctors. No insurance claim reported in any case. More than Rs. 5000 was spent in seven cases for appliance/treatment, Rs. 1000-5000 was spent in 12 cases. In one case, appliance cost was Rs 3000; in two cases, it was Rs 500-1000. Sources of appliance were private (21), Government (1) or NGO (7) and self-made (6).

## Discussion

Census data (2001) from Chandigarh had reported that 1.8% of the city population was disabled.(2) This included visual, hearing, mental, locomotor and speech disability. This information was collected during house-to-house census enumeration. Only a question on presence of a disabled person in the house and the nature of disability was asked. No further details were sought. Our study, however, has detailed information on the impact of the disability on the person as well as on the family. The higher disability rate (4.8%) reported in our study is due to the difference in the criteria used for defining disability. In fact, different surveys have yielded divergent results on prevalence of various disabilities. The NSSO survey in 1991 estimated a prevalence of 1.9% for disability (and 3% developmental delay in children). If blind and low-vision people are included, nearly 4% of India's population is disabled. In consonance with the national data, visual and locomotor disabilities were most prevalent in our study area.

Our study revealed that prevalence of disability was significantly higher in people aged 55 years or more. Similar trend is prevalent all over the world.(1) Higher disability rates among females of the study may be because of the fact that many of the families/inhabitants were migrant labour/skilled workers mainly comprising of able-bodied males from other states. No high-tech equipment or appliance was being used by disabled people in the study area. This reflects lack of market for such appliances in India i.e. a demand has not yet been created. Moreover, only one case utilized the benefit of government scheme for disabled. Even spectacles or dentures were not used by all those affected. This may be due to the high cost (affordability) or lack of suitability (appropriate appliance).

As far as effect of disability on ADL and arrangements made by the family to overcome the same is concerned, it seemed that people laid more emphasis on hygiene aspects. For example, arrangements were made by the families more frequently to take care of activities such as bathing, combing and changing clothes in many cases. On the other hand, arrangements were made less frequently to overcome restrictions in mobility. Our study revealed that disabled subjects felt more handicapped in outdoor settings such as market, road or public transport. Very little handicap was reported within the house during routine life or even during festivals, where they were duly involved in celebrations.

People need to be made aware of various government schemes available for disabled. There is also a need to popularize various gadgets available in the market for improving the quality of life of disabled people. In particular, public places (market and roads) and transport (bus and trains) need to be made more disabled friendly. This will help in reducing the degree of handicap experienced by the disabled.

## Limitation

Because of the small sample size, the results of this study should not be considered representative of all the villages of Chandigarh.
